# CGM profiling in Roux‐en‐Y gastric bypass, type 1 diabetes and healthy adults: Unmasking deviations from normoglycaemia

**DOI:** 10.1111/dom.70059

**Published:** 2025-08-26

**Authors:** Luca Cossu, Andrea Facchinetti, Lia Bally

**Affiliations:** ^1^ Department of Information Engineering University of Padova Padova Italy; ^2^ Department of Diabetes, Endocrinology, Nutritional Medicine and Metabolism (UDEM) University Hospital Bern Bern Switzerland

## 1 BACKGROUND

Bariatric surgery such as Roux‐en‐Y gastric bypass (RYGB) is a cornerstone treatment for severe obesity, driving substantial weight loss and diabetes remission.^1^ However, the benefits on diabetes are tempered by the rising recognition of post‐bariatric hypoglycaemia (PBH)—a late complication marked by hypoglycaemic episodes following rapid meal‐induced glycaemic excursions.^2^

Due to nonspecific symptoms, Continuous Glucose Monitoring (CGM)—which records interstitial glucose levels at 5‐min intervals over 10–14 days—has become a vital screening tool for PBH detection and management.

Yet interpreting CGM data in RYGB patients remains challenging: altered anatomy accelerates glucose absorption and amplifies GLP‐1 responses, yielding faster glucose peaks and lower post‐meal nadirs. While evaluation of glucose levels is often done with comparison against thresholds designed for people with diabetes, healthy non‐operated individuals may confer the more suitable comparator to define deviation from “normal”. However, such glycaemic benchmarks remain poorly characterised.

This study directly addresses this gap by contrasting ambulatory CGM profiles from RYGB, type 1 diabetes, and healthy adults, matched for sex, age, and BMI, thereby delineating deviations from normal and diabetic glycaemic physiology.

## 2 METHODS

### 2.1 Datasets and propensity score matching

We conducted a comparative analysis of CGM metrics using data from the following cohorts:

1. Cohort of 45 RYGB adults (RYGB cohort) with previously confirmed PBH (defined as symptomatic plasma or sensor glucose <54 mg/dL relieved by carbohydrate intake) who wore a masked Dexcom G6 (Dexcom Inc., San Diego, CA, USA) CGM sensor under free‐living conditions for a median of 10 days (IQR 9–30) (NCT05212207). All participants were free of medications known to interfere with glucose metabolism.
2. Healthy control cohort (healthy cohort) without diabetes or history of bariatric surgery and without any medications that might affect glucose metabolism^3^ who wore a masked Dexcom G5 for up to 10 days.
3. Cohort of adults with type 1 diabetes (T1D cohort) enrolled in the T1DExi study^4^ who wore a Dexcom G6 for up to 1 month and were treated with insulin pump therapy.

To address potential confounding by sex, age, and BMI, we performed propensity score matching, which resulted in three balanced groups (*n* = 45 each) for subsequent glycaemic analyses. To ensure balanced data availability across all cohorts, we used 10 consecutive days of CGM recording per subject with ≥70% sensor wear time.

All cohorts consisted of 36 female and 9 male participants. The RYGB cohort (age 48.8 ± 13.3 years, BMI 26.7 ± 5.2 kg/m^2^) had non‐diabetic haemoglobin A1c (HbA1c) of 5.4 ± 0.4%, as did healthy controls (48.3 ± 20.0 years, 25.5 ± 2.8 kg/m^2^) with an HbA1c of 5.2 ± 0.2%. The T1D cohort (48.1 ± 13.8 years, 27.4 ± 6.1 kg/m^2^) showed higher HbA1c (6.5 ± 0.6%), estimated from CGM‐derived glucose values using established methods.^5^ The quality of matching was evaluated using standardized mean differences (SMDs) for all covariates, with values <0.1 indicating adequate balance. All post‐matching SMDs were below this threshold, confirming that a balanced match was achieved.

### 2.2 Evaluated glucose metrics and statistical testing

From the 10‐day CGM recordings, we calculated the following metrics according to international consensus guidelines^6^: time in range (TIR, 70–180 mg/dL), time in tight range (TITR, 70–140 mg/dL), time above range (TAR, >180 mg/dL), time below range (TBR, <70 mg/dL), the time in Level 1 (L1) hypoglycaemic range (54–70 mg/dL) and the time in L2 hypoglycaemic range (<54 mg/dL), which is considered clinically significant hypoglycaemia, including in non‐diabetic individuals.^7^ Additionally, we calculated the number and the average duration of both L1 and L2 hypoglycaemic events, defined as at least three consecutive CGM data points below the hypoglycaemic threshold (54 mg/dL for L2 and 70 mg/dL for L1).^6^ These metrics were computed for: (1) the full 24‐hour period, (2) daytime (06:00–24:00), and (3) nighttime (00:00–06:00).

Depending on the normality of the data, comparisons between groups were conducted using either the unpaired *t* test (for normally distributed variables) or the Mann–Whitney *U* test (for non‐normally distributed variables). A *p*‐value of less than 0.05 was considered statistically significant. All analyses were performed using MATLAB 2024b.

## 3 RESULTS

**FIGURE 1:**
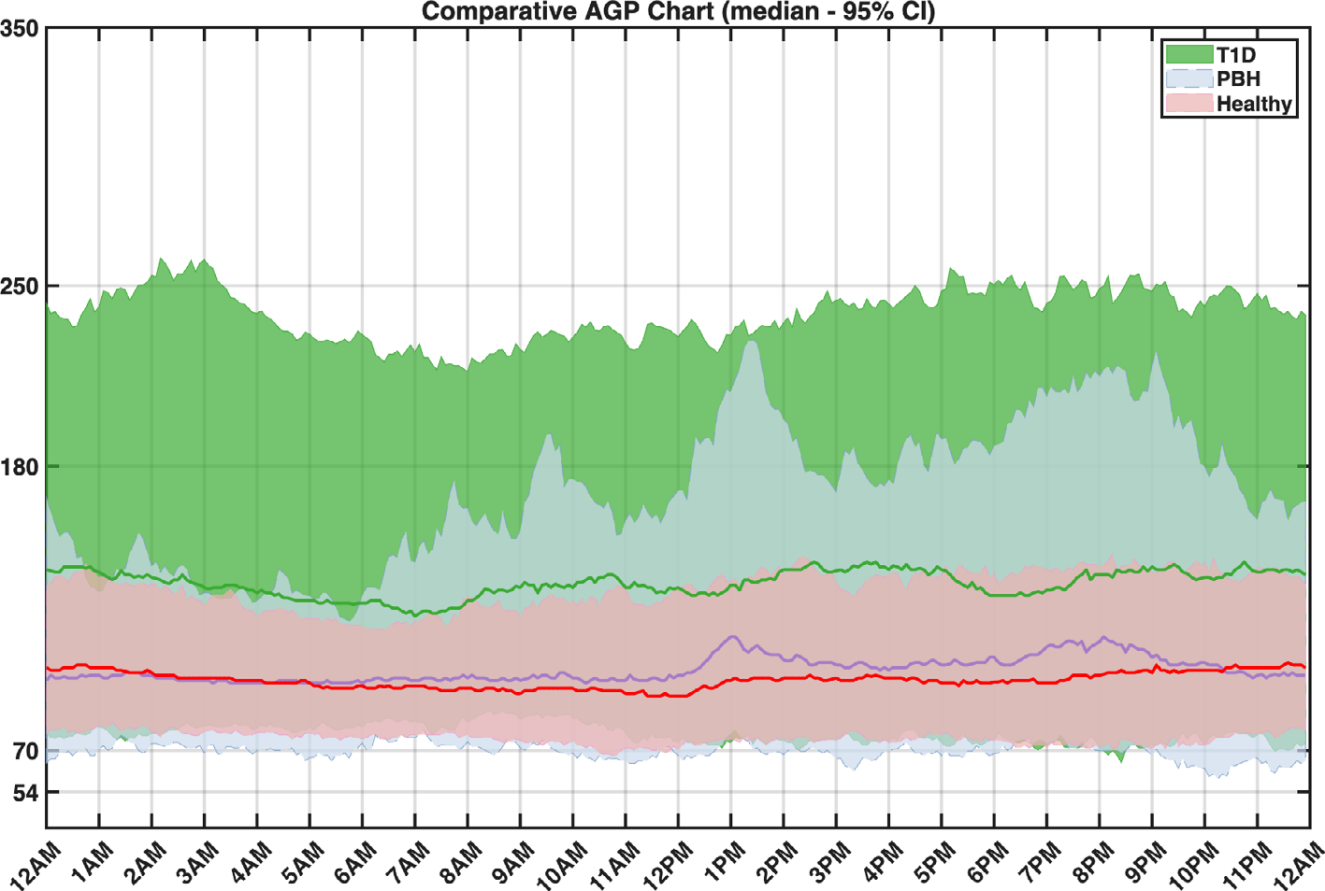
Comparative chart between the consolidated daily glucose traces of all groups. The line shows the median value during the day, while the area shows the 5th–95th percentiles of the data over the 10 days.

Quantitative results for all glycaemic metrics are presented in Table 1, including *p*‐values for comparisons between RYGB participants and both control groups (healthy controls and T1D cohort) across all analysed time periods. The 24‐h median 95% CI sensor glucose profiles by cohort are shown in Figure 1.

**TABLE 1:** CGM‐based glucose metrics across the three cohorts (RYGB, healthy, T1D) reported as median and interquartile range for the full data, for the daytime and the nighttime.

Metric	[A] Overall			[B] Daytime			[C] Nighttime		
	RYBG *n* = 45	Healthy *n* = 45	T1D *n* = 45	RYBG *n* = 45	Healthy *n* = 45	T1D *n* = 45	RYBG *n* = 45	Healthy *n* = 45	T1D *n* = 45
Mean glucose level (mg/dL)	107.7 (9.2)	99.1 (9.8)**	139.1 (19.1)++	111.0 (12.8)	99.9 (8.5)**	141.0 (22.4)++	98.9 (11.8)	98.4 (12.0)	139.3 (25.7)++
TIR (%) [70–180 mg/dL]	91.6 (7.2)	98.3 (3.0)**	78.9 (14.5)++	89.5 (7.2)	98.3 (3.8)**	76.9 (16.4)++	96.4 (5.7)	99.0 (3.0)**	80.1 (22.1)++
TITR (%) [70–140 mg/dL]	82.9 (8.6)	94.7 (5.5)**	53.2 (16.4)++	78.7 (9.9)	94.1 (5.8)**	52.3 (18.4)++	96.4 (5.7)	97.9 (5.4)*	52.3 (25.2)++
TAR (%) [>180 mg/dL]	3.5 (3.9)	0.0 (0.3)**	17.3 (13.9)++	4.5 (5.5)	0.0 (0.3)**	18.8 (13.9)++	0.1 (1.9)	0.0 (0)**	17.7 (18.5)++
TBR (%) [<70 mg/dL]	3.4 (4.5)	1.6 (3.2)**	2.1 (3.3)++	3.6 (5.3)	1.5 (3.8)**	2.1 (3.6)++	2.6 (4.3)	0.7 (2.5)**	1.6 (2.8)+
CV	29.0 (8.5)	16.4 (4.4)**	31.7 (8.3)+	30.2 (7.4)	16.7 (4.4)**	31.9 (8.1)	16.2 (9.4)	11.6 (6.4)**	30.9 (11.3)++
L1 hypoglycaemic events	12.0 (10.2)	4.0 (8.5)**	8.0 (9.25)++	10.0 (8.5)	3.0 (7.0)**	6.0 (9.0)++	2.0 (4.0)	1.0 (2.0)**	1.0 (2.2)+
L1 hypoglycaemic events duration (min)	32.0 (18.4)	32.4 (19.9)*	34.4 (18.8)+	30.1 (18.8)	31.9 (21.7)*	31.6 (15.2)+	30.0 (22.6)	30.0 (27.9)*	41.8 (32.8)+
L2 hypoglycaemic events	3.0 (4.0)	0.0 (1.0)**	1.0 (3.0)+	2.0 (4.0)	0.0 (0.0)**	1.0 (3.0)+	0.0 (2.0)	0.0 (0.0)**	0.0 (1.0)+
L2 hypoglycaemic events duration (min)	26.5 (14.0)	22.5 (15.4)	23.3 (15.4)	25.0 (14.1)	22.5 (15.0)	22.5 (12.7)	26.6 (13.1)	20.0 (15.0)**	41.6 (25.0)++
Hyperglycaemic events	12.0 (11.2)	0.0 (1.0)**	23 (10.2)++	12 (11.2)	0.0 (1.0)**	19 (9.5)+	0 (1.2)	0.0 (0.0)**	5.0 (5.0)++
Hyperglycaemic events duration (min)	32.5 (12.1)	25.0 (17.8)*	112.1 (67.7)++	32.5 (11.9)	21.6 (17.5)*	94.1 (39.8)++	26.2 (21.6)	36.6 (18.7)*	121.8 (93.1)++
Time in L1 hypoglycaemic range (%)	2.8 (3.5)	1.6 (2.6)**	1.9 (2.5)++	2.9 (4.2)	1.4 (3.6)**	1.9 (3.1)++	1.9 (2.6)	0.7 (2.5)*	1.1 (2.5)+
Time in L2 hypoglycaemic range (%)	0.6 (1.6)	0 (0.2)**	0.2 (0.6)+	0.5 (1.4)	0.0 (0.1)**	0.3 (0.7)+	0.5 (1.6)	0 (0.2)**	0.0 (0.6)+

*Note*: * and ** denote absolute SMD compared to healthy of >0.2 and >0.5, respectively; + and ++ denote absolute SMD compared to T1D of >0.2 and >0.5, respectively.

RYBG participants exhibited significantly higher average glucose levels relative to healthy controls, evident both in the overall 24‐h period (107.7 ± 9.2 mg/dL vs. 99.1 ± 9.8 mg/dL, SMD > 0.5) and during daytime hours (111.0 ± 12.8 mg/dL vs. 99.9 ± 8.5 mg/dL, SMD > 0.5), while maintaining comparable nighttime levels.

The TIR analysis revealed significantly impaired glycaemic control in RYGB participants compared to healthy controls (91.6 ± 7.2% vs. 98.3 ± 3.0%, SMD > 0.5), with particularly pronounced differences during daytime (89.5 ± 7.2% vs. 98.3 ± 3.8%, SMD > 0.5). Concurrent elevations in both time above range (TAR) and time below range (TBR) metrics underscore the unstable glycaemic profile of RYGB patients, characterised by rapid oscillations between hyper‐ and hypoglycaemic states. This metabolic lability was further quantified by significantly higher coefficients of variation (29.0 ± 8.5% vs. 16.4 ± 4.4%, SMD > 0.5), indicating substantially greater glucose fluctuations in the RYGB cohort.

The RYGB cohort experienced more hypoglycaemic episodes, with both L1 (12.0 ± 10.2 vs. 4.0 ± 8.5, SMD > 0.5) and L2 (3.0 ± 4.0 vs. 0.0 ± 1.0, SMD > 0.5) at significantly higher frequencies than healthy controls. The duration of individual hypoglycaemic episodes showed no intergroup difference. Concurrently, RYGB patients demonstrated marked hyperglycaemic excursions, exhibiting both greater incidence (12.0 ± 11.2 vs. 0.0 ± 1.0 events, SMD > 0.5) and prolonged duration (32.5 ± 12.1 min vs. 25 ± 17.8 min, SMD > 0.2) compared to controls. Compared to the T1D cohort, RYGB individuals demonstrated significantly lower mean glucose levels (107.7 ± 9.2 vs. 139.1 ± 19.1, SMD > 0.5) and more time in target and tight target ranges. RYGB individuals exhibited a significantly higher frequency of hypoglycaemic events compared to T1D individuals, both overall and during daytime periods. Consequently, cumulative time spent in both L1 and L2 hypoglycaemic ranges was substantially elevated in the RYGB compared to the T1D cohort. Conversely, the number of hyperglycaemic events was higher in T1D individuals.

RYGB individuals demonstrated significantly higher glycaemic variability than healthy controls (CV: 29.0% vs. 16.4%, *p* < 0.001). While overall glycaemic variability was comparable between the RYGB and T1D cohorts, temporal patterns differed significantly. The RYGB group exhibited pronounced variability predominantly during daytime hours, whereas T1D individuals showed more evenly distributed fluctuations across both day and night periods.

## 4 CONCLUSIONS

In this work, we compared CGM profiles of RYGB individuals with a known diagnosis of PBH with those of non‐surgical non‐diabetic controls and individuals with T1D, matched for age, sex, and BMI.

This design enabled us to distinguish RYGB‐specific glucose patterns from both normal physiology and insulin‐deficient diabetes under exogenous insulin therapy while eliminating potential confounders.

While glucose fluctuations in RYGB individuals were less pronounced than in the well‐controlled T1D cohort, they significantly exceeded those of healthy controls, with more frequent hypo‐ and hyperglycaemic events. These alterations predominantly occur during daytime hours and reflect characteristic post‐RYGB physiology around meals: accelerated intestinal nutrient absorption, amplified incretin hormone responses, and consequent insulin hypersecretion inadequately counterbalanced by glucose‐raising hormones.^8^, ^9^, ^10^

Recurrent hypoglycaemic episodes and increased glycaemic variability warrant clinical attention due to their substantial impacts on quality of life, daily functioning, and safety.^11^ When evaluating and managing these fluctuations, clinicians should target healthy population norms. This differentiation proves particularly crucial for hypoglycaemia management, as transient glucose levels <70 mg/dL may represent normal physiological variation^3^ and frequently warrant observation rather than intervention—an important consideration given the potential for iatrogenic weight gain through unnecessary carbohydrate administration.^12^ However, the current absence of standardized glycaemic targets for the RYGB population creates clinical uncertainty, underscoring the need for evidence‐based guidelines. These knowledge gaps are compounded by a limited understanding of CGM diagnostic performance and the intricate relationship between glucose dynamics and symptom manifestation in post‐bariatric patients.^13^, ^14^

While this comparative analysis provides valuable insights, several limitations warrant consideration. The modest sample size may constrain the generalisability of our findings, and the restricted CGM monitoring duration might not fully represent long‐term glycaemic variability patterns. Building on these findings, future work should incorporate more diverse cohorts, including individuals after sleeve gastrectomy and extended CGM monitoring periods, allowing for the development of stratified reference databases, accounting for age, sex, BMI and other characteristics. These efforts are particularly relevant for special populations such as pregnant individuals, whose unique physiological adaptations in glucose metabolism currently lack well‐defined CGM standards.^15^

When interpreting these results, the accuracy profile of the used CGM devices requires consideration. According to the literature, the Dexcom G5 and G6 systems demonstrate an overall mean absolute relative difference (MARD) of approximately 9% across all glycaemic ranges. However, accuracy deteriorates significantly in the hypoglycaemic range (≤70 mg/dL), with MARD increasing to 19%–21% compared with reference glucose values.^16^ This reduced accuracy at low glucose concentrations suggests that some apparent hypoglycaemic readings may represent measurement artefacts rather than true biochemical hypoglycaemia. Conversely, the Dexcom G5 and G6 were previously demonstrated to systemically over‐read glucose values in hypoglycaemic ranges, potentially leading to missed true hypoglycaemic episodes.^17^, ^18^

In the RYGB population, isolated low CGM values should therefore be interpreted with care; ideally confirmed by capillary glucose testing in both clinical and research contexts. Additionally, this analysis did not account for potentially confounding variables including dietary and physical activity behaviour, or insulin therapy characteristics in type 1 diabetes. Given that CGM metrics are substantially influenced by these factors, their omission limits the contextualisation of observed glucose profiles and necessitates prudent interpretation of the findings.

In conclusion, RYGB establishes a unique glycaemic signature differing from both normal physiology and insulin‐managed T1D. These findings highlight the need to establish comprehensive, population‐specific CGM normative values to better contextualise the metabolic consequences of bariatric surgery on glycaemic profiles.

## CONFLICT OF INTEREST STATEMENT

The authors declare no conflicts of interest.

## PEER REVIEW

The peer review history for this article is available at https://www.webofscience.com/api/gateway/wos/peer-review/10.1111/dom.70059.

## Data Availability

The data that support the findings of this study are available from the corresponding author upon reasonable request.
